# Growth Hormone/Insulin Growth Factor Axis in Sex Steroid Associated Disorders and Related Cancers

**DOI:** 10.3389/fcell.2021.630503

**Published:** 2021-03-18

**Authors:** Rachel Bleach, Mark Sherlock, Michael W. O’Reilly, Marie McIlroy

**Affiliations:** ^1^Endocrine Oncology Research Group, Department of Surgery, RCSI University of Medicine and Health Sciences, Dublin, Ireland; ^2^Academic Department of Endocrinology, Beaumont Hospital and RCSI Medical School, Dublin, Ireland

**Keywords:** cancer, IGF, sex steroids, endocrine, metabolism

## Abstract

To date, almost all solid malignancies have implicated insulin-like growth factor (IGF) signalling as a driver of tumour growth. However, the remarkable level of crosstalk between sex hormones, the IGF-1 receptor (IGF-1R) and its ligands IGF-1 and 2 in endocrine driven cancers is incompletely understood. Similar to the sex steroids, IGF signalling is essential in normal development as well as growth and tissue homoeostasis, and undergoes a steady decline with advancing age and increasing visceral adiposity. Interestingly, IGF-1 has been found to play a compensatory role for both estrogen receptor (ER) and androgen receptor (AR) by augmenting hormonal responses in the absence of, or where low levels of ligand are present. Furthermore, experimental, and epidemiological evidence supports a role for dysregulated IGF signalling in breast and prostate cancers. Insulin-like growth factor binding protein (IGFBP) molecules can regulate the bioavailability of IGF-1 and are frequently expressed in these hormonally regulated tissues. The link between age-related disease and the role of IGF-1 in the process of ageing and longevity has gained much attention over the last few decades, spurring the development of numerous IGF targeted therapies that have, to date, failed to deliver on their therapeutic potential. This review will provide an overview of the sexually dimorphic nature of IGF signalling in humans and how this is impacted by the reduction in sex steroids in mid-life. It will also explore the latest links with metabolic syndromes, hormonal imbalances associated with ageing and targeting of IGF signalling in endocrine-related tumour growth with an emphasis on post-menopausal breast cancer and the impact of the steroidal milieu.

## Introduction

The hypothalamic-pituitary (HP) axis is the central regulator of endocrine action, controlling the function of a number of endocrine glands including thyroid, adrenal, gonadal and the growth hormone (GH)/insulin growth factor-1 (IGF-1) system, which modulate a myriad of physiological processes. The HP axis integrates various stimuli, including those generated internally such as energy levels, stress, inflammation and also in response to external stimuli (dark/light cycles and temperature) (Petrescu et al., 2018) and orchestrates endocrine outputs which control numerous physiological processes. The role of endocrine hormones and growth factors are interlinked, often being involved in the same cellular functions leading to crosstalk between the pathways and demonstrating bi-functional roles. Moreover, alterations in signalling through these pathways leads to a diverse number of diseases including many cancers, neurodevelopmental disorders and metabolic syndromes, highlighting the critical importance of understanding the precise regulation of these pathways and their interconnectivity. Furthermore, sex steroids crosstalk with several growth factors such as the epidermal growth factor (EGF) (Auricchio et al., 2008), fibroblast growth factor (FGF) (Smith et al., 2002), vascular endothelial growth factor (VEGF) (Hyder, 2006), transforming growth factor (TGF), platelet-derived growth factor (PDGF) (Schmahl et al., 2008), nerve growth factor (NGF) (Luo et al., 2018), and IGF in both normal and cancerous cells (Kenney and Dickson, 1996). This occurs at a number of different levels to influence cellular processes, including the production of steroids (Schmahl et al., 2008; Luo et al., 2018). Many of these growth factors are also known to be involved in mechanisms of resistance to endocrine therapies for the treatment of breast and prostate cancer (Schiff et al., 2003). This review aims to outline the role of the IGF/IGF-1R and steroid hormone interplay during normal growth and development, followed by an in-depth look at how these pathways impact metabolism and the potential consequences of this in the development of endocrine-related cancers.

## The Hypothalamic Pituitary GH and IGF-1 Axis

The regulation of the GH/IGF-1 system is dependent on the integrity of the hypothalamus, pituitary and liver. The primary source of circulating GH is the somatotrophs of the anterior pituitary gland, however, it is also synthesised in other tissues including reproductive tissues, lymphoid tissues and the gastrointestinal tract (Harvey et al., 2000). The pulsatile secretion of GH from the anterior pituitary is carefully regulated by the stimulatory effect of hypothalamic growth hormone releasing hormone (GHRH), dietary protein and ghrelin (Milman et al., 2016) and the inhibitory effects of somatostatin and glucose. IGF-1 is a GH dependant growth factor produced in a number of tissues but predominantly in the liver in response to GH. IGF-1 circulates attached to a number of IGF binding proteins (IGFBP 1-6), which are regulated by GH to varying degrees. The most biologically important of these binding proteins is IGFBP-3 (Ballard et al., 1989). IGF-1 levels are also dependant on a number of other hormone factors including sex steroids (which may play an important role in age dependant decrease in IGF-1), thyroxine, and glucocorticoids (Sherlock and Toogood, 2007).

### Effects of Age and Gender on the GH/IGF-1 System

The GH/IGF-1 system changes over the human lifespan and these alterations are associated with, although not necessarily causative of, metabolic alterations and ageing related disease. GH pulsatile secretion is impacted by a vast array of factors including: gender, sex steroids, age, nutritional status, body composition, visceral adiposity, sleep, physical activity, and metabolic stress (Veldhuis and Iranmanesh, 1996).

During the period of increased growth associated with puberty, GH secretion rates increase resulting in a twofold to threefold increase in serum IGF-1 concentrations (Martha et al., 1992, 1996) with an associated increase in whole body protein synthesis (Mauras et al., 1996). Once growth and development are complete GH levels begin to fall. Several studies have shown a decrease in GH secretion in healthy elderly adults compared to healthy younger adults (Veldhuis et al., 1991). The age-related decrease in GH secretory burst frequency, the half-life of endogenous GH, and the daily secretory rate correlates with increasing adiposity, decreased physical performance and decreased testosterone levels (Ho et al., 1987). In men GH secretion is closely linked to serum testosterone levels, hence in individuals with primary hypogonadism the replacement of testosterone increases serum GH and IGF-1 significantly (Veldhuis et al., 1997).

The effect of age on spontaneous GH secretion is less pronounced in pre-menopausal women (Weltman et al., 1994) with secretion remaining relatively stable until after the menopause, when GH levels fall significantly. GH secretion differs considerably between men and women. Young women have approximately a twofold to threefold increase in GH serum concentration production compared to age matched males (van den Berg et al., 1996). It is now well recognised that estrogen reduces the hepatic production of IGF-1 in response to GH (Ho et al., 2006).

### IGF Signalling

The IGF signalling network consists of IGF-1R, IGF-2R, and the insulin receptor (IR). IGF-1R and the IR are classified as receptor tyrosine kinases and share a similar structure. Ligands involved in the IGF signalling pathway include IGF-1 and IGF-2. IGF-1 has highest affinity for the IGF-1R (Steele-Perkins et al., 1988) with much lower affinity for IGF-2R and the IR. IR and IGF-1R display approximately 50% sequence homology (Ullrich et al., 1986). Although they can mediate control of many of the same intracellular pathways with many interconnected physiologic functions, the biological outputs influenced can also be exceptionally distinct as proven though the use of knockout models (Liu et al., 2000; Kitamura et al., 2003; Coan et al., 2008). Furthermore, Cai et al. (2017) proved by mutational analysis that the intracellular domain of the receptors regulates differential gene expression patterns. Normal growth and development are dependent on IGF signalling and perturbations are associated with dwarfism (Lin et al., 2018) and acromegaly (Sata and Ho, 2007), whereas the IR is more associated with the regulation of metabolic processes. However, the considerable overlap of functions between these receptors is exemplified in breast cancer where it has been suggested that inhibition of both IGF-1R and IR may be required for effective antitumour response (Fagan et al., 2012).

As well as the hypothalamic pituitary GH axis, IGF-1 secretion is controlled by autocrine/paracrine signals in peripheral tissues. Interestingly, the paracrine versus endocrine sources of IGF-1 can impact its function. Paracrine IGF-1 has a greater effect on mammary gland branching than IGF-1 from endocrine sources (Richards et al., 2004). Furthermore, the complexity of IGF signalling is enhanced by the myriad of proteins which are activated downstream of it such as the Ras/Raf/MAPK and PI3K/AKT signalling pathways. These pathways regulate numerous biological processes, alterations in which, are known contributors to carcinogenesis. Additional complexity arises from IGF-1 stimulated signalling exerting differential effects in the mammary gland depending on whether it is pre-pubertal or pubertal, with switching between the activation of PI3K/Akt and Ras/Raf/MAPK signalling pathways (Tian et al., 2012).

### Insulin-Like Growth Factor Binding Proteins

The IGF signalling pathway is influenced by a number of different factors. In order to gain more insight into the activity of this pathway studies have also looked at the levels of its regulatory proteins, the IGFBP family (Renehan et al., 2004). IGF-1 bioavailability in the circulation and activity at a tissue level is modulated by its association with six IGFBPs (IGFBP1-6). They can stimulate and inhibit IGF signalling by regulating its half-life, clearance, and modulating receptor interactions. IGF-1 is released from IGFBPs by mechanisms involving extracellular matrix (ECM) binding or proteolysis in the linker domain (Argente et al., 2017). The majority of IGF-1 in circulation is bound to IGFBPs with a low amount of free ligand present (Brahmkhatri et al., 2015). The IGFBP family members share a conserved structure, however, they differ in their functional motifs. Furthermore, IGFBP-2 (Azar et al., 2014), 3, 5 (Schedlich et al., 2000), and 6 (Iosef et al., 2008) contain a nuclear localisation sequence. IGFBPs are also found to be able to induce effects independently of IGF-1/IGF-1R. For example, IGFBP5 is known to interact with cell surface proteins which consequently increases local concentrations of IGF-1, therefore enhancing binding with IGF-1R within the vicinity (Jones et al., 1993). The role of IGFBPs in human health has not yet been fully elucidated. Combinational knockout of IGFBP3, 4, and 5 leads to a reduction in growth and also decreased fat and adipocyte size (Ning et al., 2006). However, evidence suggests that there is some redundancy between the IGFBPs and loss of only one could be compensated for by others. This highlights the importance these proteins are likely to play in the regulation of normal physiology.

### Interconnectedness of IGF and Sex Steroids Network During Development and Ageing

#### Estrogens

**FIGURE 1:**
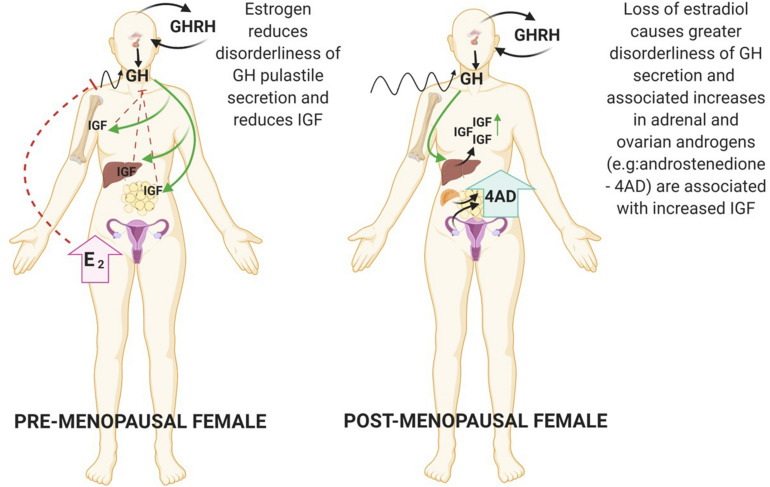
During the female lifespan sex steroid levels impact the pulsatile secretion of GH. In the premenopausal state high E_2_ reduces disorderliness of GH secretion. Reduction in E_2_ due to ovarian shutdown during menopause accompanies increases in GH pulsatile disorderliness. Created with BioRender.com.

In adulthood, estrogen is the primary sex-steroid agonist of GH secretion in both women and men (Veldhuis et al., 2005, 2006). Estrogens are synthesised from androgen substrates via the aromatase enzyme expressed in the brain, skin, ovary, adipose, bone and adrenal cortex. In breast cancer IGF-1 has been shown to increase aromatase activity (Su et al., 2011). The action of estradiol is primarily directed by interaction with the estrogen receptor (ER) nuclear receptors (alpha and beta) in peripheral target tissues further modulated via feedback by GH and IGF-1 (Veldhuis et al., 2006). Hence, whilst estrogen exerts central stimulatory effects on the GH/IGF axis, localised estrogen synthesis in peripheral tissue is inhibitory (Birzniece et al., 2012) (Figure 1). Interestingly, there have been mixed reports on the impact of HRT on IGF-1 concentrations in post-menopausal women, confounded by the different formulations (estrogen or estrogen plus progestin) and modes of delivery with oral estradiol decreasing IGF-1 concentrations and the androgenic synthetic progestin causing an increase (Campagnoli et al., 1995; Biglia et al., 2003). Indeed, in the post-menopausal woman loss of estradiol coincides with increased disorderliness of GH secretion and a diminished negative feedback control of IGF secretion.

#### Androgens

Although androgens are known to stimulate the GH/IGF-1 axis in men there is scant information in the literature about the impact of androgens on the GH/IGF-1 axis in women, which may be a significant factor especially during the post-menopausal period. This is an interesting point when you consider that in prostate cancer it has been shown that estrogen can substitute to upregulate IGF-1R when androgen levels are low (Genua et al., 2009). It is generally accepted that there is an age-related decline in androgens in women due to ovarian failure and involution of the adrenal zona reticularis. However, recent data suggest that 11-oxygenated androgens may not decline with age, which may be relevant here (Lasley et al., 2011; Turcu et al., 2017). How these weaker androgens may impact metabolism is not understood but their levels do coincide with the development of metabolic syndrome in post-menopausal women and those suffering from polycystic ovary syndrome (PCOS) (Azziz et al., 1998; Stefanska et al., 2015; Kempegowda et al., 2020). Utz et al. (2008) took a more focused look at the impact of obesity on the GH/IGF axis, they reported that androgens can maintain elevated IGF peripherally in the absence of estrogen driving GH secretion in obese, post-menopausal women. Whilst DHEA-S levels are the highest in circulation, they identified androstenedione as the most prevalent circulating androgen with a binding affinity for its cognate receptor. Whilst this is by no means a potent androgen they encouraged further investigation into the role of androstenedione as a moderator of the GH-IGF-1 axis. Specifically, they hypothesised that elevated androgens in overweight, postmenopausal women may preserve endogenous IGF even in the absence of GH stimulation.

In men, testosterone concentrations positively correlate with the regularity of GH secretion, with concentrations of both hormones, diminishing with increasing age (Veldhuis and Iranmanesh, 1996). It has been widely reported that decreasing levels of testosterone via the natural ageing process result in increased levels of visceral fat and the development of metabolic syndrome, and importantly this is apparent even in non-obese individuals (Kupelian et al., 2006; Brand et al., 2011).

## GH/IGF and Sex Steroid Signalling Impact Metabolism

**FIGURE 2:**
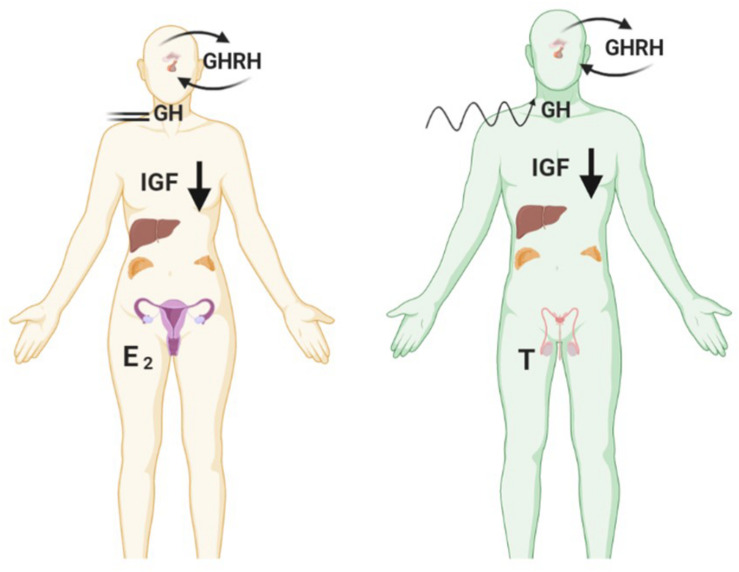
Fasting reduces levels of IGF-1 in both genders but causes opposing effects on pulsatile GH secretion. Female GH levels become constrained in contrast to greater disorderliness of pulses in males. Created with BioRender.com.

Studies have shown that nutritional status is a strong determinant of IGF gene expression, not only in liver, but also in other tissues; with fasting shown to reduce serum and tissue IGF-1 levels (Lowe et al., 1989), although there appears to be gender specific ramifications for GH secretion under fasting conditions (see Figure 2). Of interest, increased adrenal androgen production is associated with IGF-1 levels only in females, potentially due to subsequent aromatisation and stimulation of the HP-somatotrophic axis (Veldhuis et al., 1997; Guercio et al., 2002, 2003). Further adding to the complexity it is now acknowledged that sex steroids may impact metabolism through non-genomic action independent of classical steroid receptor activation which is an area that requires further elucidation (Liu and Mauvais-Jarvis, 2009; Wong et al., 2010; Navarro et al., 2016). Many diseases associated with metabolic dysregulation are linked with the perturbation of sex hormones, examples of which are outlined in the following sections.

### Type 2 Diabetes Mellitus

As alluded to earlier, the importance of the HP axis in the regulation of somatotrophic growth and steroid production and associated feedback undergoes significant changes with age. An insightful study by Rettberg et al. (2014) highlighted this by showing loss of ovarian hormones causes a reduction in glucose uptake by the brain. The authors suggest this may be attributed to myriad impairments in glucose transport and handling within neurons which ultimately results in reduced mitochondrial function (Ding et al., 2013). It is also evident that these changes in glucose metabolism are not restricted to the brain but have a knock-on effect in whole body energy storage, which manifests as increased adiposity (specifically visceral) in postmenopausal women. Whilst there is a paucity of information with regards how androgens impact metabolism and adiposity via alterations in IGF in females, there are some studies which indirectly point to a role for androgens as mediators of type 2 diabetes mellitus (T2DM). IGF signalling is linked to metabolic dysfunction with equivalently low androgen levels in males and excess androgens in females leading to type 2 diabetes and metabolic dysfunction (Navarro et al., 2015). Postmenopausal women with impaired glucose tolerance have higher androgen activity than women with normal glucose tolerance (Larsson and Ahren, 1996). Furthermore, clinical studies looking at the impact of HRT on the development of insulin resistance and T2DM showed that the homeostatic assessment of insulin resistance (HOMA-IR) score was greatly reduced in women taking hormone replacement (Salpeter et al., 2006). Importantly, this seems very much dependent upon the type of steroid in the formulation with studies in primates indicating that only estrogen could restore insulin sensitivity whereas medroxyprogesterone acetate (MPA) caused increased adiposity and reduced insulin sensitivity (Shadoan et al., 2003, 2007). Since adipose tissue expresses androgen receptor (AR) as well as ER alpha and beta we cannot discount the role that these androgens are playing in regulation of adipogenesis and metabolism particularly in the post-menopausal woman. While there are not many studies evaluating this in men there have been reports of the development of metabolic syndromes manifesting during the initial stage of insulin resistance. Moreover, both insulin and IGF-1 signalling could play an essential role in driving prostate cancer growth (Yanase et al., 2017; Drincic et al., 2018).

### Polycystic Ovary Syndrome

Polycystic ovary syndrome may provide useful clinical insights into the relationship between androgen excess, insulin signalling and metabolic dysfunction. PCOS is a common chronic health condition affecting up to 10% of all women, and is defined by androgen excess, anovulation and often polycystic ovarian morphology on imaging (Rotterdam ESHRE/ASRM-Sponsored PCOS Consensus Workshop Group, 2004). It has traditionally been perceived as a predominantly reproductive disorder, but it is increasingly clear that PCOS is a lifelong chronic metabolic disorder of women (Randeva et al., 2012). Population data shows that women with PCOS are at a 2–4-fold increased risk of developing T2DM than the background age- and BMI-matched female population, and that onset precedes diagnosis of T2DM in non-PCOS women by 4 years (Rubin et al., 2017). Androgen excess is a cardinal clinical feature of PCOS, and correlates closely with the severity of metabolic dysfunction, including insulin resistance, T2DM, and non-alcoholic fatty liver disease (NAFLD) (O’Reilly et al., 2014; Kumarendran et al., 2018).

Androgen excess is likely to be directly complicit in metabolic dysfunction in PCOS. Serum testosterone concentrations predict development of hyperglycaemia in population studies (O’Reilly et al., 2019); *in vitro*, androgen excess induces peripheral insulin resistance and pancreatic β-cell insulin hypersecretion in both female human and mouse cell culture models, predisposing to onset of β-cell failure and T2DM (Navarro et al., 2018). Adipose tissue is also a key target organ of androgen action; prenatally androgenised female mice have aberrant adipose tissue function (Roland et al., 2010), while locally generated androgens in female adipose tissue may drive *de novo* lipogenesis and adipocyte hypertrophy (O’Reilly et al., 2017). The net effect of these changes is to fuel systemic insulin resistance and hyperinsulinemia, which in turn drive androgen generation in the ovaries and peripheral tissues (Poretsky et al., 1999).

Hyperinsulinemia augments growth hormone receptor signalling, and increases hepatic IGF-1 production. Limited human *in vivo* data have identified subtle disturbances in the growth hormone-IGF-1 axis in women with PCOS, however studies to date have not elucidated if these changes are linked to hyperinsulinemia, obesity or androgen excess (Wu et al., 2000; de Boer et al., 2004). Women with PCOS are at a significantly increased risk of endometrial carcinoma (Barry et al., 2014); while this has traditionally been attributed to the endometrial hyperplasia and dysplasia in the context of chronic amenorrhoea, increased endometrial expression in women with PCOS of genes associated with the insulin signalling pathway (IGF-1, IGFBP1, and PTEN) hints at a role for IGF-1 and insulin resistance in this process (Shafiee et al., 2016). Data on the risks of other gynaecological or non-gynaecological malignancies, including breast, in PCOS are ambiguous to date, predominantly due to heterogeneity in diagnostic criteria for PCOS and the lack of large-scale prospective data. Given the high population prevalence of PCOS, urgent further studies are needed to understand the complex associations between androgen metabolism, insulin signalling, metabolic risk and malignancy in this patient cohort.

### Sarcopenia, Hypogonadism, and the IGF1 Axis

Sarcopenia is the age-related loss of muscle that leads to frailty in the elderly, diminishes their ability to lead active lives, makes them vulnerable to falls and is very detrimental to their quality of life. There are a wide range of endocrine factors that impact muscle mass and function, in particular IGF-1 signalling and androgen anabolic action and efforts to elucidate these are ongoing.

The anabolic properties of GH suggest that the manipulation of the GH/IGF-1 axis may provide a possible therapeutic option for the treatment of many of the adverse changes which occur with ageing and in particular sarcopenia. Studies in ageing populations have used GH alone or in combination with sex steroids or physical training and have examined the effect upon a number of variables including body composition, muscle strength, bone mineral density and physical performance (Sherlock and Toogood, 2007). The results of these studies have been inconsistent and there appears to be a close interplay between GH treatment/exercise and sex steroid therapy on the improvement of muscle mass/strength in sarcopenia with most studies showing that one intervention alone is less likely to lead to increase in muscle mass/strength (Taaffe et al., 1994; Papadakis et al., 1996; Lange et al., 2000; Blackman et al., 2002; Giannoulis et al., 2006). The use of GH in elderly patients (particularly in those with no evidence of GH deficiency) is not advised given the metabolic complications and also the concern regarding cancer risk (Sherlock and Toogood, 2007).

Male androgen deficiency is associated with an accelerated reduction in muscle mass and strength. Men undergoing androgen deprivation therapy for prostate cancer lose significant muscle protein content within 6 weeks of induction of hypogonadism (Lam et al., 2019). The advent of selective androgen receptor modulators (SARMs) promises significant therapeutic potential to ameliorate the sarcopenic effect of androgen deprivation therapy in the future. SARM therapy as an adjuvant to androgen deprivation therapy in prostate cancer may protect against the adverse musculoskeletal, metabolic and neuro-cognitive impacts of hypogonadism induced by GnRH agonists and other therapies for prostate cancer, while at the same time inhibiting prostate cancer tissue proliferation and growth (Chisamore et al., 2016).

To date, there are no convincing clinical data linking hypogonadism with disturbances in growth hormone secretion or responsiveness to stimulation as a potential exacerbating factor in hypogonadism-induced sarcopenia. In one small proof-of-principle physiology study, untreated hypogonadal men had an intact IGF1 response to GH stimulation, and this response was unchanged by cross-over treatment with both testosterone and dihydrotestosterone administered as transdermal gel (Gleeson and Shalet, 2009). Supporting this finding, acute hypogonadism induced by GnRH agonist therapy in healthy men did not result in blunting of the GH response to dynamic stimulation, although basal GH secretion was marginally reduced (Veldhuis et al., 2009). On the basis of these limited data, it appears unlikely that the GH-IGF1 axis plays a direct role in the mediation of loss of muscle mass associated with hypogonadism.

### Insulin-Like Growth Factor Signalling and Adipose Tissue

Although often overlooked, adipose tissue is an endocrine organ which plays a very important role in the secretion of many substances such as steroid hormones and growth factors. Adipose tissue is also a target for the actions of growth factors and hormones. GH and IGF-1 are involved in regulating adipocyte differentiation and proliferation and furthermore, sex hormones influence adipose tissue in numerous different ways such as gene expression and function (Chang et al., 2018). A very interesting study by D’Esposito et al., found that adipocytes from obese individuals had two fold higher levels of IGF-1 release than from lean individuals. Additionally, co-culturing adipocytes with MCF7 breast cancer cells resulted in enhanced growth (D’Esposito et al., 2012). Huang et al. (2013), found that AR knockout bone marrow stromal cells have greater adipocyte formation that their wild-type counterparts. Further studies to explore the mechanism promoting adipogenesis revealed that AR knockout decreased IGFBP3 expression which allowed IGF to activate the Akt signalling pathway.

A recently published study reported that periodic fasting or a fasting-mimicking diet can enhance the anti-cancer activity of anti-estrogen therapies by lowering the circulating levels IGF-1, insulin and leptin and consequently inhibiting the AKT mTOR pathway (Caffa et al., 2020). Conversely, hyperinsulinemia and insulin resistance are associated with increased cancer mortality in both obese and non-obese individuals (Tsujimoto et al., 2017). An important consideration therefore is the type of adipose tissue with visceral adipose deposits appearing to drive metabolic perturbations more so than their subcutaneous counterparts. This is particularly interesting when you consider the role of estrogens and androgens as evident in observed gender differences in adiposity.

## The GH/IGF-1 System and Cancer Risk

For several decades there has been an accumulation of data from epidemiological studies, basic science research and studies related to patient groups with altered levels of GH/IGF-1 which has suggested that the GH/IGF-1 system may be associated with either tumourigenesis or more aggressive behaviour in cancers (Holly et al., 1999). Laron syndrome is associated with insensitivity to GH and results in obesity and very low levels of IGF-1 in serum. However, affected individuals are reported to have reduced risk of developing cancer (Werner et al., 2019). In acromegaly (a condition with elevated GH and IGF-1 concentrations due to a pituitary tumour) (Dineen et al., 2017) some but not all studies have suggested an increased risk of developing cancer (Sherlock et al., 2010; Dal et al., 2018; Dworakowska and Grossman, 2019). Collectively, these data support epidemiological and experimental evidence of a role for GH and IGF-1 in the development of cancer.

### Insulin Growth Factors and Cancer

Although oncogenic mutations frequently initiate cancer development, the growth and expansion of tumours can also be mediated by growth factors. Cells that have undergone oncogenic transformation often display overexpression of growth factors and dysregulation of signalling pathways downstream of these growth factors. Local production of growth factors in normal tissue is limited and therefore competition for availability coupled with a balance of pro versus anti-growth signals in the local environment restrains cell growth in a controlled manner. Growth factors are not simply involved in driving growth of the tumour, they can also impact the tumour microenvironment (Zhang et al., 2010), and cancer-cell de-differentiation (Nakano et al., 2019). Growth factors have been found to be involved in all steps of tumour invasion and metastasis (reviewed by Witsch et al., 2010). Additionally although signalling via growth factor receptors can be oncogenic the same receptors can also drive apoptosis within cancer cells (Ali et al., 2018).

IGF mutations do not occur frequently in cancer indicating that it is often not an initial driver of tumourigenesis (Simpson et al., 2017). In the MSK-IMPACT clinical sequencing cohort of over 10,000 cancer patients 2.4% had a genetic alteration in IGF-1R (Cerami et al., 2012; Gao et al., 2013; Zehir et al., 2017). However, artificial overexpression of IGF-1R *in vitro* does result in malignant transformation (Kaleko et al., 1990). IGF-1R activity and expression are frequently increased in malignant tumours showing it to certainly play a role in the progression of tumourigenesis. It has been reported that increased IGF-1R activity in cancers may occur secondary to the loss of tumour suppressor genes such as *TP53, BRCA1*, von-Hippel Lindau protein and Wilms’ tumour-1 (Werner, 2012). Kruger et al., also reported that IGF-1R activation rather than IGF-1R overexpression is sufficient to induce downstream activation of the MAPK/PI3K signalling pathways and overcome tamoxifen treatment in breast cancer (Kruger et al., 2020).

Of the IGFBP family of proteins IGFBP3 has been extensively investigated and is the most frequently linked to the pathogenesis of cancer (Johnson and Firth, 2014; Cai et al., 2020). However, it has been associated with both pro-tumourigenic and anti-tumourigenic functions due to its ability to inhibit or enhance IGF actions. A collection of evidence now also points to IGF/IGF-IR-independent actions of IGFBP-3. Depending on cell type this has revealed both tumour suppressing and tumour promoting effects. Through its interaction with proteins located on the cell surface and within the cell, IGFBP-3 is involved in several biological processes that are independent of IGF-1/IGF-1R. It is known to interact with nuclear hormone receptors which include the vitamin D receptor (Moreno-Santos et al., 2017) and the retinoid X receptor (RXR) (Schedlich et al., 2007). IGFBP3 was found to mediate anti-tumour activity through its interaction with the RXR. By modulating the translocation of the RXR binding partner (orphan nuclear receptor Nur77), from the nucleus to the mitochondria it inhibits cell growth and induces apoptosis (Lee et al., 2005).

### Insulin-Like Growth Factor and Endocrine-Related Cancer

**FIGURE 3:**
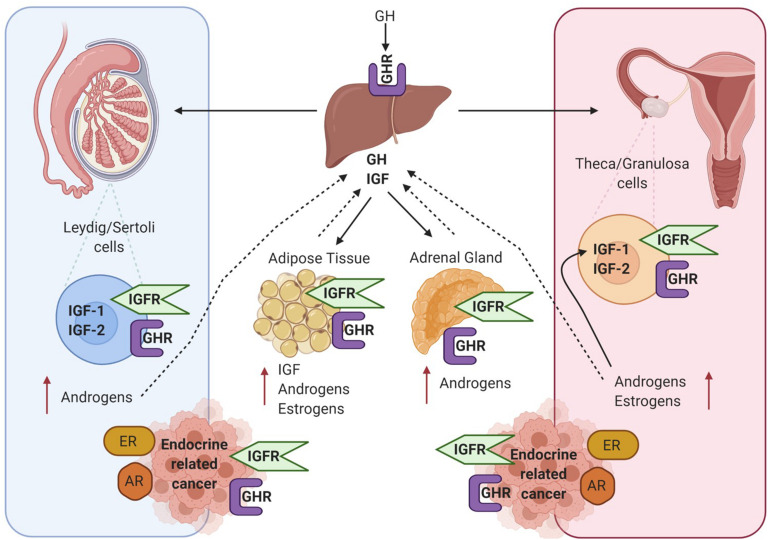
Diagram showing crosstalk between the hypothalamic pituitary GH and IGF-1 axis and endocrine organs – testis in men, ovaries in woman and adipose tissue and adrenal gland in both sexes. All these tissue express the GHR and IGF1R and are also involved in the production of estrogens and androgens. GH, IGF and sex steroid autocrine and paracrine signalling can influence the development of endocrine-related cancers such as breast and prostate. Created with BioRender.com.

Many studies have demonstrated that in the absence of IGF-1 there is an impairment of gonadal steroidogenesis (Baker et al., 1996). As shown in Figure 3 there are many levels of crosstalk between the hypothalamic pituitary GH and IGF-1 axis, and endocrine organs. This in turn can have an impact on the development and progression of endocrine-related tumours which express receptors for GH, IGF, androgens and estrogens.

**FIGURE 4:**
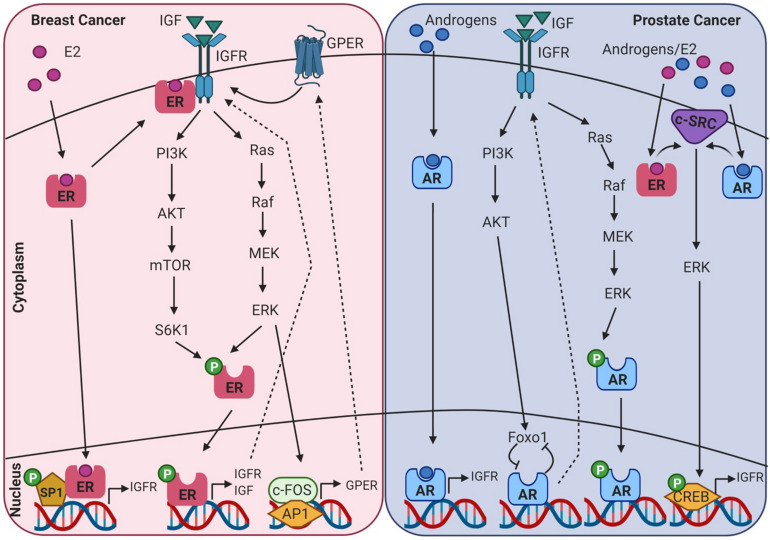
Diagram showing intracellular crosstalk between sex steroid receptors and IGF pathways in breast and prostate cancer. This illustrates the convergence of genomic and non-genomic signalling as mediators of transcriptomic gene expression in endocrine-related cancers. Created with BioRender.com.

AR, ER, and IGF signalling are involved at multiple ontogenetic stages of life. Crosstalk between the IGF signalling system and the steroid hormone receptor superfamily in endocrine-related cancers may be mediated through genomic or non-genomic signalling cascades that can be ligand dependent or independent, as displayed in Figure 4. This can occur at many levels, such as at the cell surface by phosphorylation of the IGFR, through crosstalk with cell signalling cascades, and ultimately converging at the level of transcriptional regulation. Crosstalk between IGF-1 and ER is known to regulate gene expression in breast cancer cells, but the underlying mechanisms are not fully understood. This is further confounded by ligand-dependent or ligand-independent activation of ER. Cascio et al. (2007) found that in MCF7 breast cancer cells estradiol and IGF-1 differentially regulate ER transcription at ERE and AP-1 sites. FOXA1 is a pioneer factor which is a protein that facilitates transcription factor – DNA binding. Nuclear steroid receptor DNA binding and transcriptional activation is hugely dependent upon the presence of the pioneer factor FOXA1 which co-ordinates ER and AR binding (Bernardo and Keri, 2012). FOXA1 is also a known mediator of IGF-1 activity and genes that are regulated by IGF-1 are enriched for FOXA1 binding sites. In addition, IGF-1 stabilises FOXA1 protein expression (Potter et al., 2012). IGFBP3 has also been identified as a gene target of FOXA1 and has been shown to be involved in the regulation of cell proliferation in prostate cancer cells (Imamura et al., 2012).

### Prostate Cancer

There are conflicting reports as to the influence of serum IGF-1 levels and the risk of developing prostate cancer. Some studies have shown an increased risk (Chan et al., 2002) while other studies have shown no correlation (Chan et al., 2002). IGF-1 has been shown to initiate growth response in both androgen dependent and androgen-independent prostate cancer cell lines (Orio et al., 2002). Moreover, it is known to enhance AR transactivation in low androgen environments (Orio et al., 2002). An interesting point to note is that the level of androgens present directly influence Src/ERK activation in a parabolic manner. In low physiological androgen levels (0.01–10 nmol/L) the pathway is activated, however, in higher concentrations (100 nmol/L) it is inhibited (Unni et al., 2004). In prostate cancer cells Foxo1 is recruited by ligand activated AR to the promoter of AR target genes. Here it interferes with AR-DNA interactions. Activation of the IGF1/insulin-PI3K/Akt pathway and the subsequent phosphorylation of Foxo1 ameliorates this inhibitory effect. This also results in a positive feedback loop between IGF-1 and AR as androgens activating AR stimulate IGF-1R expression (Fan et al., 2007). The close association between IGF-1 and AR is further evident with two androgen response elements (ARE) located in the upstream promoter of IGF-1 (Wu et al., 2007). In prostate cancer cells androgens have been shown to upregulate the expression of IGF-1R and as a consequence results in increased proliferation and invasion when stimulated with IGF-1 (Pandini et al., 2005). Furthermore, IGF and AR signalling pathways display a feed-back loop and can regulate expression of each other. However, mutational analysis demonstrated that androgen driven upregulation of IGF-1R was not driven by AR genomic activity but involved the activation of the Src-extracellular signal-regulated kinase pathway (Pandini et al., 2005).

### Breast Cancer

In hormone-dependent breast cancer cells, the IGF-1R and ERα are frequently co-expressed. IGF-1R mRNA and protein levels are higher in luminal (ER+ve) cell lines compared to non-luminal cell types (Iida et al., 2019). It has been shown that at least part of estrogen induced IGF-IR gene transcription in breast cancer cells is controlled by interactions between ERα and the transcription factor Sp1 (Sharon et al., 2006). Many observational studies have been conducted to investigate if there is a link between IGF-1 signalling and breast cancer risk. Most but not all prospective studies have reported a positive association between IGF-1 and breast cancer risk particularly in ER positive breast cancer however the influence of age and menopausal status remains ambivalent as highlighted in results of studies summarised in Table 1 (risk highlighted in bold) (Rinaldi et al., 2006; Schernhammer et al., 2006; Endogenous Hormones and Breast Cancer Collaborative Group et al., 2010; Kaaks et al., 2014; Murphy et al., 2020).

**TABLE 1:** Observational studies of IGFl levels and breast cancer risk.

**Risk**		**Breast cancer subtype**	**IGFBP3 concentration stat us**	**Menopausal**	**References**
IGF1	HR per 5 nmol/l increment = **1.11** (95% CI, 1.07–1.16; *p* < 0.0001)	Not reported	Not reported	No influence on association	Murphy et al. (2020)
IGF1-genetically- predicted	Mendelian randomisation analyses per 5 nmol/l increment OR = **1.05** (95% CI, 1.01–1.10; *p* = 0.02)	ER positive – OR = 1.06	No association with breast cancer risk	Not reported	Murphy et al. (2020)
		ER negative – OR = 1.02			
IGFl	Highest and lowest quartiles, OR = **1.34** (95% CI, 1.00–1.78; *p* = 0.03)	ER positive – OR = 1.41	Not reported	50 years or older	Kaaks et al. (2014)
		ER negative – OR = 1.16			
IGFl	Highest vs. lowest quintile, OR = **1.28** (95% CI, 1.14–1.44; p < 0.0001)	CR positive – OR 1.38	No influence on association	No influence on association	Endogenous Hormones and Breast Cancer Collaborative Group et al. (2010)
		ER negative – OR 0.80			
IGF1	Highest vs. lowest quintile, OR = **1.38** (95% CI, 1.02–186; *p* = 0.01)	Not reported	OR 1.44	Age > 50 at diagnosis	Rinaldi et al. (2006) (EPIC)
IGFl	Top vs. bottom quartile, RR **0.98** (95% CI, 0.69–1.39)	Premenopausal:	No association with breast cancer risk	No influence on association	Schernhammer et al. (2006) (Nurses’ Health Study II)
		ER negative – RR 1.25			
		ER positive – RR 1.14			

*OR = odds ratio, RR = relative risk, CI = confidence interval, HR = hazard ratios.*

A pooled data analysis of 17 prospective studies showed a higher odds ratio for IGF-1 in ER positive breast cancer (Endogenous Hormones and Breast Cancer Collaborative Group et al., 2010). Additionally, IGF-1 and estradiol has been shown to co-regulate the expression of a set of genes associated with breast cancer outcome (Casa et al., 2012). It is widely accepted that earlier onset of menarche is associated with increased breast cancer risk. Supporting evidence which links hormones and IGF to risk in breast cancer was found in a longitudinal study of 183 girls, where-in association of menarche and breast cancer risk may be due to estrone-to-androstenedione ratio and IGF-1 concentrations (Biro et al., 2021). It is interesting to note that it is the activity rather than the level of expression of IGF-1R that may be more relevant to these effects. In a study of 438 breast cancer patients, activated IGF-1R/IR as indicated by phosphorylation status was predictive of reduced survival (Law et al., 2008). One of the mechanisms involved in the crosstalk between the ER and the IGF-1R involves E_2_ induced phosphorylation of the IGF-1R and subsequent activation of ERK (Kahlert et al., 2000). Phosphorylation of IGF-1R and subsequent activation of downstream signalling cascades were also found to contribute to tamoxifen resistance and drive cell proliferation in breast cancer (Kruger et al., 2020). The IGF-1/IGF-1R axis can also induce phosphorylation of ER through ribosomal S6 kinase 1 (S6K1), downstream of the PI3K/AKT/mTOR pathway which results in the upregulation of IGF-1, IGF-1R and other ER target genes (Becker et al., 2011). In mammary epithelial cells, constitutively active IGF-1R induces cells to undergo epithelial to mesenchymal transition which is associated with increased migration and invasion (Kim et al., 2007). Also it has been found that a loss of E-cadherin such as is observed in invasive lobular breast carcinoma (ILC) results in increased expression and activity of the IGF-1R pathway (Nagle et al., 2018). Moreover, a recent report by Kang et al., identified a pro-tumourigenic transcriptomic phenotype in normal mammary tissue associated with increased future risk of breast cancer. This microenvironment was characterised by an 80% increase in adipocyte nuclei, larger adipocytes and activation of gene sets associated with adipogenesis including IGF-1 (Kang et al., 2020). When you look to the less well studied G protein estrogen receptor (GPER) in human breast tumour samples, GPER expression correlates with IGF-1R expression (Pandini et al., 2005). IGF-1 can also regulate GPER expression in ER+ breast cancer cells through the IGF-IR/ERK/c-fos/AP1 transduction pathway (De Marco et al., 2013).

### Clinical Trials Targeting IGF Overview in Endocrine-Related Cancers

As IGF-1R has been implicated in several cancer types it has garnered a great deal of interest as a therapeutic target. Pre-clinical assessments presented it as a very promising target and subsequently led to substantial clinical efforts to develop drugs against it. Unfortunately, the clinical trials conducted have failed to produce the anticipated benefits to patients and disappointingly there are now very few active or recruiting clinical trials targeting IGF in cancer. By searching the clinicaltrials.gov database one can see that there are currently no therapeutic agents targeting IGF in phase four clinical trials for endocrine-related cancers.

Initially monoclonal antibodies (mAB) targeting IGF-1R such as dalotuzumab (MK-0646), ganitumab (AMG479), cixutumumab (IMC-A12), and figitumumab (CP-751871) were investigated. These did not show therapeutic benefit, some reasons postulated for this include crosstalk between the IGF-1R and other growth factor pathways such as the IR (Buck et al., 2010) and feedback signalling resulting in increased release of growth hormone. Other confounding issues were side effects such as hyperglycaemia and metabolic disruption (Robertson et al., 2013; Gradishar et al., 2016). The methods for overcoming this feedback mechanism included the use of bispecific antibodies and other small molecule tyrosine kinase inhibitors (TKIs). XGFR is a bispecific antibody that was developed to target EGFR and IGF-1R and has shown some promise in pre-clinical studies of metastatic osteosarcoma (Gvozdenovic et al., 2017). XGFR was developed as a single chain Fab heterodimeric bispecific IgG (OAscFab-IgG) antibody which targets IGF-1R and EGFR by containing one binding site for each target antigen (Schanzer et al., 2014). As for the more general TKIs the sequence homology between IGF-1R and IR-A/B kinase domains presented a major problem and therefore side effects were of great concern (Guha, 2013). None have proceeded to clinical development.

The IGF-signalling system has long been an area of therapeutic interest in the treatment of breast cancer which was further supported by the findings of both the European Prospective Investigation into Cancer and Nutrition (Kaaks et al., 2014) and the Endogenous Hormones and Breast Cancer Collaborative group (Endogenous Hormones and Breast Cancer Collaborative Group et al., 2010). Consistent with these studies, in March 2020 the largest single study on the relationship of IGF-1 and breast cancer was published. From the results obtained the authors concluded that there is a probable causal relationship between circulating IGF-1 concentrations and breast cancer regardless of menopausal status. Interestingly this study also reported a positive association between genetically predicted IGF-1 concentrations and breast cancer risk however this was only in ER+ tumours (Murphy et al., 2020). Over the past 20 years there have been approximately 15 clinical trials targeting IGF in breast cancer listed as terminated or completed on the clinicaltrials.gov database. None of these have progressed any further and therefore have not contributed to the clinical management of the disease. That said, a significant point to note is that drugs targeting IGF did work well in some patients, but the limiting factor was the ability to enrich for these patients using validated biomarkers. Therefore, the important questions to be asked here are; why targeting IGF hasn’t been significantly beneficial in the clinical setting? What have we learnt from these clinical trials? And are we any closer to identifying a robust biomarker for IGF targeted therapies in cancer?

A more recent approach being investigated is the use of therapies targeting the IGF ligands directly to reduce their bioavailability. Dusigtumab (MEDI-573) and Xentuzumab (BI-836845) are dual IGF-I/IGF-II neutralizing antibodies which have now entered clinical trial. Xentuzumab has a binding affinity for both IGF-1 and IGF-2. The advantage of this drug is that it does not target the isoform B of the IR which is involved in glucose metabolism and therefore does not induce the hyperglycaemia and metabolic toxicity observed with IGF-1R targeted therapies or TKIs (de Bono et al., 2020). Xentuzumab has already been evaluated in combination with enzalutamide for the treatment of castration resistant prostate cancer (NCT02204072), however it failed to improve progression free survival over enzalutamide alone. The authors stated that the treatment was given to patients at an advanced stage of their disease as they had already failed docetaxel and abiraterone; they concluded that investigation at an earlier point in their treatment course is warranted (Hussain et al., 2019). *In vitro* and patient-derived xenograft (PDX) models of prostate cancer have since found evidence that PTEN and the abnormally spliced ligand independent androgen receptor variant 7 (AR-V7) could have potential use as biomarkers for response to this combination of therapy. In prostate cancer cell models xentuzumab failed to inhibit AKT phosphorylation in PTEN-null cells. Regarding AR-V7, its expression was increased in the xentuzumab + enzalutamide group compared with the enzalutamide-only group (Weyer-Czernilofsky et al., 2020). Xentuzumab is also being investigated in a phase 2 trial as a combination with everolimus and exemestane in patients with HR+/HER2- advanced or metastatic breast cancer with recruitment ongoing and expected completion of the trial in 2022 (NCT03659136). Another approach is to target IGFBP proteins, but in order to do so we need a more comprehensive understanding as to their role in both normal physiology and cancer. This is particularly relevant with the emergence of evidence suggesting that IGFBPs may bind directly to multiple growth factors (Wang et al., 2020). An innovative approach to using IGF signalling pathways to target cancer is to use them to direct therapies to the tumour, for example, they can be conjugated to nanoparticles to enhance targeting and penetration of the tumour (Zhou et al., 2015).

Of key importance to the advancement of IGF targeting therapies in cancer is a comprehensive understanding of its role under normal physiological conditions. In normal prostate cells IGF-1 maintained differentiated cellular characteristics, however in prostate cancer cells it induced a mesenchymal phenotype (Mansor et al., 2020). While the IGF pathway has been well studied in cell models of endocrine-related cancers we are undoubtedly lacking clinical evidence. The eligibility criteria for enrolment into clinical trials targeting the IGF pathway was not specific enough to identify patients most likely to have a positive clinical response. However, all is not lost in the potential for use of IGF therapies in the treatment of cancer. A recent study highlighted the benefit of a dual IGF-1R/IR inhibitor linsitinib to restore sensitivity to endocrine therapy in breast cancer. Although a lot of the evidence is based on cell models they did find that p-IGF-1R/IR positivity in ER+ breast cancer is associated with reduced benefit from adjuvant tamoxifen in postmenopausal patients. Interestingly, they also report that in cell lines, stimulation rather than overexpression of IGF-1R is driving tamoxifen resistance to be abrogated by linsitinib. This highlights the need for biomarkers that indicate activity of the IGF signalling pathway (Kruger et al., 2020).

## Summary

The interconnected IGF/IGFR and sex steroid signalling pathways play crucial roles in normal growth and development and their perturbation is often associated with diseases of metabolism. What remains to be understood is how age, estrogen and visceral adiposity jointly regulate the secretion of GH in ageing humans and how this may differ between genders. Both androgens and estrogens influence IGF-1 release, and their respective signalling pathways are intertwined. This has important implications for the many cancers that are linked to the development of metabolic syndrome in ageing adults such as tumours of the breast and prostate. Furthermore, as outlined previously IGF signalling is integrated with nutritional status and metabolism and now more than ever a focus on lifestyle interventions such as diet and exercise is necessary. With future studies in this area a greater understanding of IGF pathway activation will certainly lead to a resurrection of IGF targeted therapies in cancer but critically this time with an ability to identify the patients who will most benefit from it.

## Author Contributions

RB and MM conceived and co-wrote the main manuscript. MO’R and MS contributed to the sections with particular clinical relevance to endocrine disorders. All authors contributed to the article and approved the submitted version.

## Conflict of Interest

The authors declare that the research was conducted in the absence of any commercial or financial relationships that could be construed as a potential conflict of interest.
